# The use of a bed with an insulating system of electromagnetic fields improves immune function, redox and inflammatory states, and decrease the rate of aging

**DOI:** 10.1186/s12940-020-00674-y

**Published:** 2020-11-23

**Authors:** E. Díaz-Del Cerro, C. Vida, I. Martínez de Toda, J. Félix, M. De la Fuente

**Affiliations:** 1 Department of Genetics, Physiology and Microbiology (Unity of Animal Physiology), Faculty of Biology, Complutense University of Madrid (UCM), José Antonio Novais, 12, 28040 Madrid, Spain; 2 Institute of Investigation 12 de Octubre (i+12), Madrid, Spain; 3 Applied Molecular Medicine Institute, School of Medicine, Universidad San Pablo CEU, CEU Universities, Madrid, Spain

**Keywords:** Electromagnetic fields, Immune functions, Oxidative-inflammatory state, Biological age

## Abstract

**Background:**

The immune system, as a homeostatic system, is an excellent marker of health and has also been proposed as an indicator of the rate of aging. The base of the age-related changes in the immune system, “immunosenescence”, is oxidative-inflammatory stress. Studies have shown that long-term exposure to electromagnetic fields (EMFs) produced by technology causes inhibitory effects on the immune response and increases oxidation. The aim of the present study was to investigate the effects of resting on an EMF-insulated system on several immune functions, the oxidative-inflammatory state and subsequently the rate of aging (biological age).

**Methods:**

Several immune functions, in peripheral blood neutrophils and mononuclear cells, of 31 volunteers were analyzed before and after 2 months of using a bed with the patented HOGO system, which insulated participants against EMFs. Several oxidative and inflammatory parameters, in whole blood cells, were also studied. The biological age was calculated using a mathematical formula, which was based on several immune function parameters. A placebo group of 11 people using beds without that property were used as a control.

**Results:**

The results showed a significant improvement of immune functions and antioxidant and anti-inflammatory defenses after using the HOGO system for 2 months. In addition, a decrease in oxidants and pro-inflammatory compounds, a lowering of oxidative damage in lipids and in DNA as well as a reduction of calculated biological age was also observed. The placebo group did not show any changes.

**Conclusions:**

In conclusion, 2 months of resting on a bed insulated from EMFs demonstrates improvement in immune function, oxidative-inflammatory state and biological age.

## Background

The immune system, which protects an organism from infectious agents and malignant cells, is one of the regulatory systems, and consequently has an important function in the maintenance of homeostasis and health [1].

With the aging process the immune system undergoes a gradual deterioration referred to as “immunosenescence”, which has been suggested as being involved in the rate of aging [2]. Moreover, several functions of immune cells have been proposed as markers of the rate of aging, allowing the determination of the biological age of each individual [3].

In addition, immune cells need to produce free radicals and other oxidant and inflammatory compounds in order to perform their defensive functions, but an excess of them can cause alterations in biomolecules, since these cells are very sensitive to oxidative damage [4]. Thus, the balance between oxidants and pro-inflammatory compounds and antioxidant and anti-inflammatory markers is essential to preserve the functional capacity of immune cells and, therefore, the health of the organism and the achievement of successful aging [2]. In fact, the oxidative-inflammatory stress situation of immune cells in an individual has been shown to be directly related to his/her immunosenescence and life span [5, 6].

It is known that the maintenance of health, and consequently the rate of aging, depends not only on genes, but also largely on lifestyle factors [2]. In this context, on the one hand, many studies have tried to find lifestyle strategies that, improving immune function, allow the aging process to slow down and to avoid the appearance of pathologies and alterations associated with age, such as nutrition, physical and mental activity, positive social environment and the control of stress [7–10]. On the other hand, several studies have been carried out to detect the detrimental effects of different present day lifestyle habits on the rate of aging and concretely on oxidative-inflammatory stress and immune function. Thus, we are going to concentrate on the effect of electromagnetic fields (EMFs) exposure during sleeping time.

During aging, the ability to initiate and maintain sleep is decreased [11]. Several studies have shown a direct association of the amount and quality of sleep with health and general well-being [12]. Sleep is necessary for efficient cognitive, metabolic and immune functions. In fact, the link between the immune system and sleep has been known since the 1970s [13]. Nocturnal sleep has a homeostatic role in the regulation of immunity, in both innate and adaptive responses [14]. The number of leukocytes achieves a maximum in the evening or early night and then declines to reach a minimum in the early morning [15, 16]. Moreover, although the increase of inflammatory cytokines is necessary to achieve sleep [17], the restriction and deficiency of sleep have been related to a systemic inflammation [14]. Since increasing evidence suggests that chronic sleep disruption has detrimental effects on health and life expectancy [18], another relevant strategy to slow down aging could be to achieve a good quantity and quality of sleep.

Over the past 35 years, numerous papers have addressed the deleterious effects of exposure to electromagnetic fields (EMFs), which have largely increased in the last decades as a consequence of the expanding use of technology, on health and longevity [19, 20]. In this context, it is essential to consider the effects of EMFs disrupting normal sleep [21], since they affect the amount and quality of sleep, leading to accelerated aging, and, consequently, decreasing human longevity [20].

With respect to the effects of EMFs on the immune system, several studies have observed that they can affect immune organs and innate and adaptive immunity [22–25]. Thus, the deleterious effects on immune system function after exposure to EMFs have been addressed, although these depend on the quality, duration, frequency and power density of EMF exposure and the specific state of the cell [26]. In fact, after exposure to a variety of EMFs, a decrease has been found in total lymphocytes and in T-lymphocyte CD3 and CD4 and CD8, NK cells [27–29] as well as lower blood cytotoxic activity of NK lymphocytes [29].

In addition, an abundant number of studies have shown that EMFs in their entire frequency spectrum (low to high) induce an increase in reactive oxygen species (ROS) and oxidative stress in many experimental rodent models as well as in humans [26, 30].

In this context and due to the fact that EMF exposure at night has more severe effects on health in comparison to EMF radiation during the day time [21], the aim of the present study is to investigate the positive effects on the immune system, oxidative-inflammatory states and biological age of using a bed with a system that avoids EMFs, for 2 months.

## Material and methods

### Study groups and experimental design

The participants were 43 men and women (50 ± 12 years old) with similar characteristics regarding social class (upper middle), education (the majority with Bachelor’s degree) and lifestyle, information obtained after completing a survey about eating habits, smoking, degree of education and social class. They were divided into three groups: an experimental group using HOGO system beds, which avoided EMFs (*n* = 20), of which 9 were men and 11 women, at an age of 52 ± 7, a placebo group (*n* = 11), 6 women and 5 men with an age of 49 ± 5 years, which slept on normal beds, but visually identical to those with the HOGO system. A third group was a “topper” group (*n* = 12), 10 women and 2 men, who slept on beds made of non-natural materials but with a topper with the patented system to avoid EMFs.

**Fig. 1:**
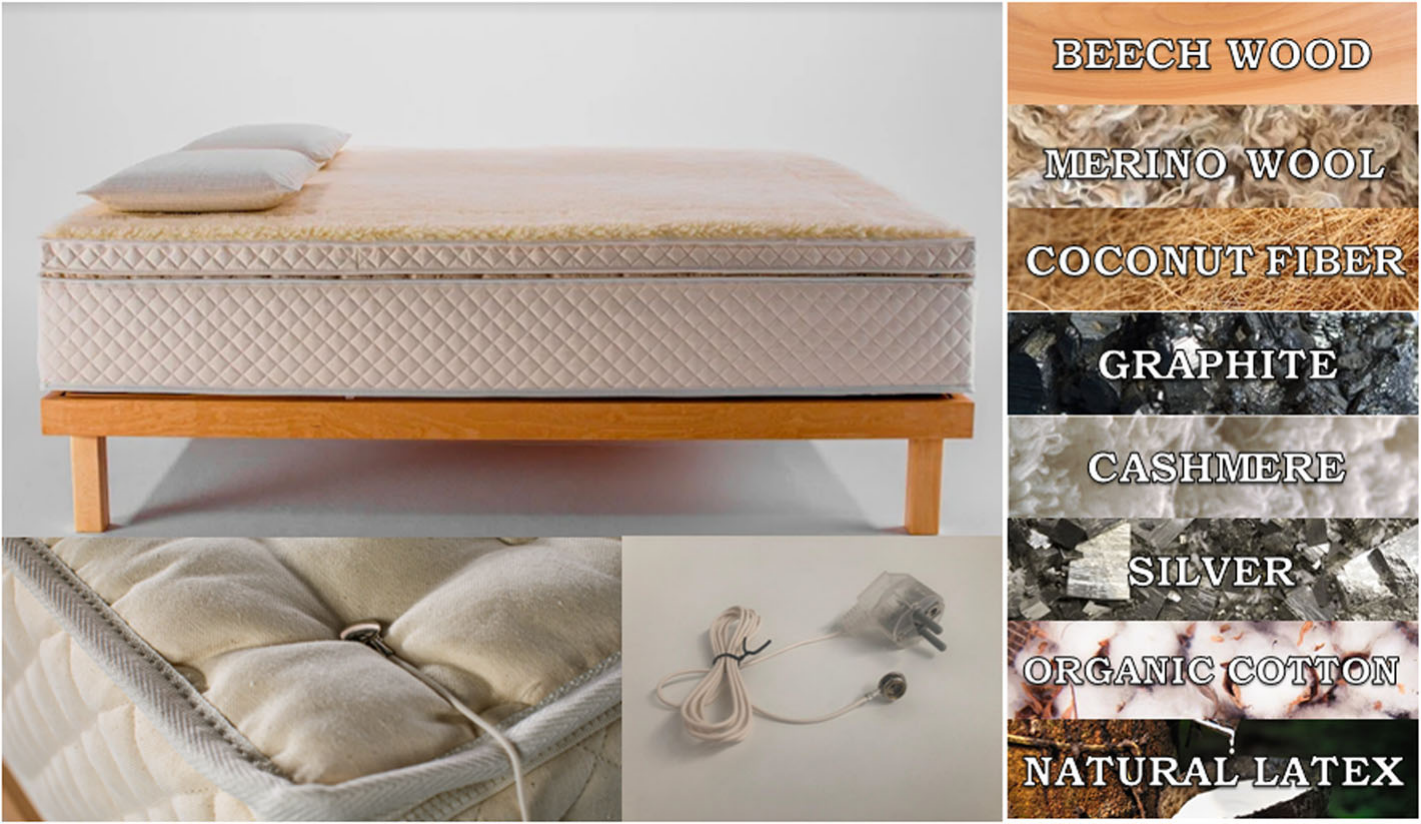
HOGO rest system structure and materials

HOGO beds (HOGO company) consists of an articulated box spring, customized, with pivoting and adaptation system for each part of the body, laminated and vaporized beech wood, natural varnishes and natural rubbers as part of the laminate support. The mattress and topper are composed of milk from the *Hevea brasiliensis* tree, coconut fiber, Merina wool, Caschemer, bamboo, silver, graphite, organic cotton, and patented technology for the removal of EMFs. The pillow is composed of milk from the *Hevea brasiliensis* tree, woven with graphite and silver maya and patented technology for the removal of EMFs. Finally, the blanket is made of Merina wool tested and flaky to prevent allergies and improve perspiration and oxygenation. The HOGO rest system is connected to the building grounding system for the removal of residual and accumulated EMFs in the person during the day, creating a similar situation than a Faraday cage. The plug is made of plastic and rubber so as not to be a conductor of the electricity of the plugs. It includes a no-return diode for safety (Fig. 1). Normal beds are visually equal but without patented technology for the removal of EMFs and made with not-100%-naturals materials and with metallic springs.

The volunteers largely consisted of friends or acquaintances of HOGO workers, who were randomly assigned a standard bed or the HOGO rest system. Most of the participants in this study are people who were given, free of charge, a standard bed (Placebo bed) or a bed with the HOGO system (the patented system to avoid EMFs and the natural materials). Only 5 individuals from the experimental group purchased the HOGO rest system. In addition, the standard bed or placebo and HOGO beds were visually identical, so volunteers could not distinguish them. The participants received an explanation of the experimental design and the possible beneficial effects of sleeping on a bed with the characteristics of a system that isolates from EMFs of the environment and that is made of natural materials. Moreover, they also knew that they could receive a bed with the HOGO system or a placebo one. In none of the cases the volunteers were compensated with money, and they received the standard bed or the HOGO system to use at home. Both standard beds and HOGO beds were installed in the room of each volunteer, without making any other changes in this room. Thus, they continued with their usual lifestyle. The electromagnetic fields were measured throughout the room (walls, furniture, floor, windows...) before and after plugging in the system in the sleeping area. Electromagnetic fields in low frequencies were measured using ME3830B analyzer (Gigahertz solutions) with a frequency range of 16 Hz-100 KHz, magnetic flux density (one-dimensional): 1–1999 nT and electric field strength: 1–1999 V/m. High-frequency electromagnetic radiation was measured by HF35C analyzer (Gigahertz solutions) with a frequency range of 800 MHz - 2.7 GHz and a power flux density of 0.1–1999 μW/m^2^. Thus, the low frequency electric fields measurements in the room were 984.7 ± 275.3 mV before plugging in the HOGO system, and 34.9 ± 12.7 mV, after. High frequency electric field measurements were not relevant since these can only be detected if homes are near municipal WI-FI or mobile phone facilities.

According to Building Biology Evaluation Guidelines for Sleeping Areas SBM-2008 [31], frequency range limits are shown in Table 1.

**Table 1:** Frequency range limits in sleeping areas

	No concern	Slight concern	Severe concern	Extreme concern
**Electric Fields**				
With grounding cable (V/m)	< 1	1–5	5–50	> 50
Potential-free (V/m)	< 0.3	0.3–1.5	1.5–10	> 10
**Magnetic Fields** (nT)	< 20	20–100	100–500	> 500
**High frequency** (μW/m^2^)	< 0.1	0.1–10	10–1000	> 1000

Before and after resting for 2 months on these beds, 12 mL of blood were obtained from each participant between 9:00–10:00 a.m.

Exclusion criteria were: 1) Severe and unstable medical conditions, or a history of chronic diseases; 2) aphasia, confusion, or psychiatric comorbidity; 3) taking medications, such as anti-inflammatory agents, muscle relaxants, corticoids and antidepressants; 4) any previously diagnosed sleep diseases, including narcolepsy, periodic limb movement disorder, obstructive sleep apnea and Parkinson’s disease related sleep disorders; 5) previous surgery; 6) pregnancy; 7) electrohypersensitivity and 8) non-cooperation during the evaluation.

This study was approved by the Ethical Committee of Clinical Research of the University Hospital 12 de Octubre of Madrid (N° CEI 18/221).

### Collection of human blood samples and isolation of lymphocytes and neutrophils

Peripheral blood samples (12 mL) were collected using vein puncture and sodium citrate-buffered Vacutainer tubes (BD Diagnostic, Spain), between 9:00 to 10:00 a.m. to avoid circadian variations in immune parameters. Neutrophil and lymphocyte cells were isolated from whole blood following a previously described method [32, 33], in which blood samples were centrifuged at 700 g for 60 min on a density gradient using 1.119 and 1.077 density Hystopaque (Sigma-Aldrich, Spain) for neutrophil and lymphocyte separation, respectively. Cell viability was checked by the trypan blue exclusion test. Viable cells were over 98%. Collected cells were counted and adjusted to 10^6^ neutrophils or lymphocytes per mL of Hank’s solution or RPMI 1640 medium.

### Immune parameters

#### Adherence

Adherent capacity of neutrophils and lymphocytes was measured following a method, previously describe [32], which mimics in vitro the adherence of immune cells to the vascular endothelium. Briefly, 1 mL of whole blood (diluted 1:1 with Hank’s medium) was placed in a Pasteur pipette in which 50 mg of nylon fiber was packed to a height of 1.25 cm. After 10 min, the effluent had drained by gravity. The percentage of adherence or adherence index (A.I.) was calculated as follows:

$$ A.I.=100-\frac{\mathrm{neutrophils}\ \mathrm{or}\ \mathrm{lymphocytes}\ \mathrm{per}\ \mathrm{mL}\ \mathrm{of}\ \mathrm{effluent}\ \mathrm{samples}}{\mathrm{neutrophils}\ \mathrm{or}\ \mathrm{lymphocytes}\ \mathrm{per}\ \mathrm{mL}\ \mathrm{of}\ \mathrm{or}\mathrm{iginal}\ \mathrm{samples}}\ x\ 100 $$

#### Chemotaxis

The chemotaxis or induced mobility of neutrophils and lymphocytes was evaluated according to the method previously described [33]. Aliquots of 300 μL of the neutrophil or lymphocyte suspension were deposited in the upper compartment of a Boyden chamber separated by a filter of nitrocellulose (Millipore, Mildford, MA, USA) of 3 μm pore diameter. Formyl-met-phe-leu (Sigma, St. Louis, MO, USA), a chemoattractant agent, was put in the lower compartment at 10^− 8^ M to induce chemotaxis. After 3 h of incubation at 37 °C and 5% CO_2_, the filter was fixed (methanol 50% and ethanol 75%) and stained (azur-eosin-methylene blue solution, GIEMSA, PANREAC). The chemotactic index (C.I.), representing the total number of neutrophils or lymphocytes counted by optical microscopy (immersion objective) on one-third of the lower face of the filters, was calculated.

#### Phagocytosis

Phagocytosis of inert particles (latex beads) was assayed in neutrophils following a method previously described [33]. Aliquots of 200 μL of neutrophil suspension were incubated on migration inhibition factor (MIF) plates (Sterilin, Teddington, UK) for 30 min and the adherent monolayer was washed with PBS (phosphate buffer saline) at 37 °C, and 20 μL latex beads (1.09 μm diluted to 1% PBS, Sigma- Aldrich) were added. After 30 min of incubation, the plates were washed, fixed (methanol 50%) and stained with azur-Eosin-Methylene Blue solution and the number of particles ingested by 100 neutrophils (Phagocytosis Index), and the percentage of neutrophils that ingested at least one particle (Phagocytic Efficacy), were determined by optical microscopy (immersion objective).

#### Natural killer cytotoxicity

The natural killer (NK) cell cytotoxicity was evaluated following an enzymatic colorimetric assay (Cytotox 96 TM Promega, Boeringher Ingelheim, Germany) based on the determination of lactate deshydrogenase (LDH) released by the cytolysis of target cells (line of cells K562 from a human lymphoma), using tetrazolium salts [33]. Target cells were seeded in 96-well U-bottom culture plates at 10^4^ cells/well in RPMI medium without phenol red. Effector cells (lymphocytes) were added at 10^5^ cells/well, the effector/target rate being, 10/1. The plates were centrifuged at 250 g for 5 min to facilitate cell to cell contacts and then they were incubated for 4 h. After incubation, LDH activity was measured by addition of the enzyme substrate and absorbance recording at 490 nm. The results were expressed as the percentages of lysis of tumour cells (% lysis), which were determined with the following equation:

$$ \%\kern0.35em lysis=\frac{\mathrm{E}-\mathrm{ES}-\mathrm{TS}}{\mathrm{M}-\mathrm{TS}}\ x\ 100 $$

where E is the mean of absorbance in the presence of both effector and target cells, ES the mean of absorbance of effector cells incubated alone, TS the mean of absorbance of target cells incubated alone and M the mean of maximum absorbance after incubating target cells with lysis solution.

#### Lymphoproliferation

The proliferation capacity of lymphocytes was evaluated by a standard method, previously described [33] in suspensions of lymphocytes. The assay was assessed in both basal and stimulated conditions by mitogen (Phytohaemagglutinin (PHA), 1 μg/mL (Sigma-Aldrich, Madrid, Spain). The suspensions of mononuclear leukocytes were adjusted to 10^6^ lymphocytes/mL of RPMI (Gibco) supplemented with gentamicin (1 mg/mL, Gibco) and 10% fetal bovine serum (FBS) (Gibco), previously inactivated by heat (30 min at 56 °C). Aliquots of 200 μL were dispensed in plates of 96 wells (Costar, Cambridge, MA, USA) and 20 μL of phytohemagglutinin (PHA, Flow) to 20 mg/L was used as mitogen. 20 μL of RPMI supplemented medium were added to controls. After 48 h of incubation, 0.5 μCi/well ^3^H-thymidine (Dupont, Boston, MA) was added, followed by another 24 h of incubation. The cells were harvested in a semiautomatic harvester and thymidine uptake was measured in a beta counter (LKB, Upsala, Sweden) for 1 min. The results were expressed as ^3^H-thymidine uptake (cpm), both in basal and PHA stimulated cells.

### Biological age calculation

The biological age of each volunteer was calculated using a mathematical model, which takes into account five immune function parameters (neutrophil chemotaxis and phagocytosis, lymphocyte chemotaxis, natural killer activity as well as lymphoproliferation in response to mitogen stimulus), constructed throughout multiple linear regression [3, 34, 35].

### Oxidative stress parameters

Blood was centrifuged at 1300 g for 20 min. Then, plasma and total blood cells were separated. Total blood cell pellets were reconstituted with RPMI+ medium and frozen at − 80 °C until use.

#### Catalase activity

Cell pellets were diluted in the reaction buffer and the catalase activity was measured by the catalase fluorometric detection kit (ADI-907-027, ENZO). Fluorescence was measured at 530 nm excitation and 590 nm emission. The results were expressed as units (U) of CAT activity/mg protein.

#### Glutathione peroxidase (GPx) activity

Cell pellets were diluted in oxygen-free phosphate buffer 50 mM. Then they were sonicated and supernatants were used for the enzymatic reaction together with cumene hydroperoxide as a substrate (cumene-OOH) as was described previously [34]. Oxidation of NADPH was measured at 340 nm. The results were expressed as units (U) of GPx activity/mg protein.

#### Glutathione concentrations

Cell pellets were diluted in phosphate buffer 50 mM and EDTA 0.1 M, pH 8. Then, they were sonicated and supernatants were used for the quantification of both reduced (GSH) and oxidized (GSSG) glutathione by o-phthalaldehyde (OPT) at pH 12 and pH 8, respectively, resulting in the formation of a fluorescent compound, as previously described [34]. Fluorescence was measured at 350 nm excitation and 420 nm emission. Results were expressed as nmol of GSSH and GSH per milligram of protein. Moreover, the GSSG/GSH ratio was calculated for each sample.

#### Hydrogen peroxide (H_2_O_2_) concentrations

Cell pellets were diluted in phosphate buffer 50 mM, pH 6.0. Hydrogen peroxide content was measured by a colorimetric kit (ADI-907-015, ENZO). The results were expressed as ng/mg protein.

#### Lipid peroxidation (Thiobarbituric acid reactive substances (TBARS) assay)

Lipid peroxidation was evaluated using a commercial kit (BioVision, Mountain View, CA, USA), which measures the reaction of malondialdehyde (MDA) with thiobarbituric acid (TBA) and the MDA-TBA adduct formation [36]. Samples were resuspended in lysis buffer with the antioxidant butylated hydroxy-toluene (BHT) (0.1 mM) to prevent further formation of MDA during the preparation of the sample or during the heating step. Then, they were sonicated and centrifuged at 13000 g for 20 min. Later, The 200 μL of supernatants from each sample were added to 600 μL TBA, and incubated at 95 °C for 60 min. Samples were cooled in ice for 10 min, and 300 μL of n-butanol were added (Sigma-Aldrich) to create an organic phase in which the MDA molecules were to be placed. Samples were centrifuged and 200 μL of upper organic phase were collected and dispensed into a 96-well microplate for spectrophotometric measurement at 532 nm. Results were expressed as nmol TBARS/mg protein.

#### DNA damage: quantitation of 8-hydroxy-2′-deoxyguanosine

The 8-hydroxy-2′-deoxyguanosine (8-OHdG) was evaluated in samples of total blood cells using the commercial kit DNA Damage ELISA (ADI-EKS-350, ENZO). Absorbance was measured at 450 nm. Results were expressed as ng8-OHdG/mL.

### Protein concentrations

The protein content of the same samples was evaluated following a bicinchoninic acid protein assay kit protocol (Sigma-Aldrich, Madrid, Spain).

### Cytokine measurements

Blood samples were incubated for 4 h without (basal) or with 10 μL of the mitogen lipopolysaccharide (LPS) (stimulated) (250 ng/mL, Sigma-Aldrich). Later, samples were centrifuged at 1300 g for 20 min and plasma was collected. The basal and stimulated release of IL-1β, IL-6, TNF-α, IL-4 and IL-10 were measured simultaneously in these supernatants by multiplex luminometry (Beadlyte human multiplex cytokine detection system, MHYSTOMAG-70 K, Upstate, Millipore). Results were expressed as pg/mL.

### Statistical analyses

The data were analyzed using SPSS 21.0 (SPSS Inc., Chicago, IL, USA). The Kolmogorov–Smirnov test was used to test for normality and the homogeneity using the Levene test. Quantitative variables are presented as mean (x) ± standard deviation (SD), whereas categorical variables are presented as number and %. Comparisons between the groups were made by the independent-samples t-test according to the compatibility of the data with normal distribution and comparisons between results of a same group were made by the dependent-samples t-test. The difference between proportional variables was calculated by the chi-square test. All tests were two-tailed, with a significant level of α = 0.05.

## Results

In the following results, men and women data from each experimental group were not separated because no statistically significant differences were found due to sex in any of the parameters investigated.

**Fig. 2:**
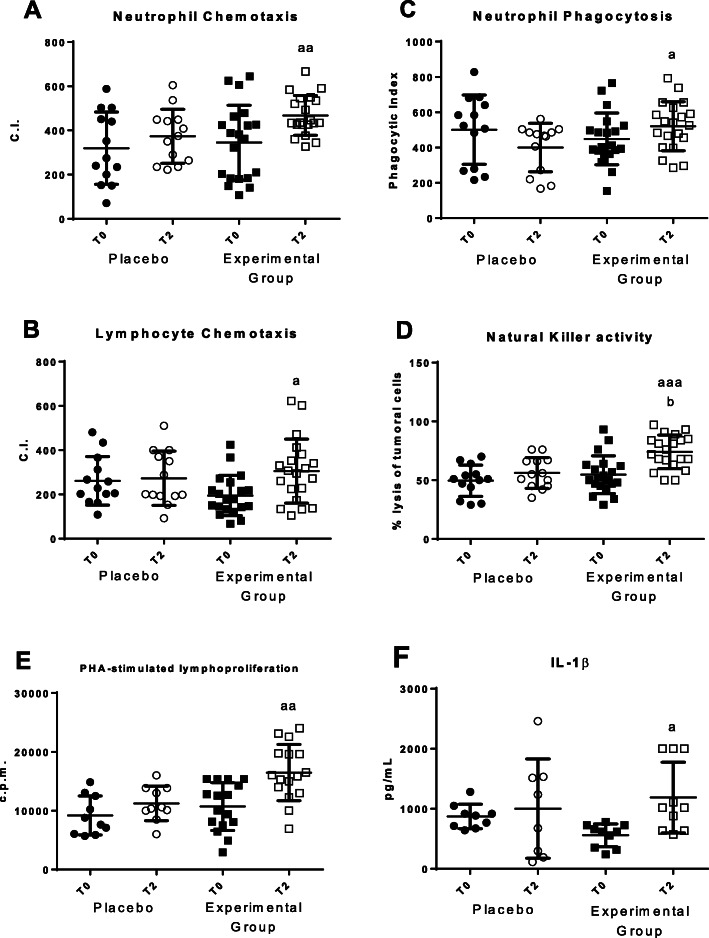
Changes in immune functions in peripheral blood leukocytes before (T0) and after 2 months (T2). **a** Chemotaxis Index (CI) of neutrophils (number of neutrophils on the filter); **b** Chemotaxis Index (CI) of lymphocytes (number of lymphocytes on the filter); **c** Phagocytic Index (PI) (number of latex beads ingested per 100 neutrophils); **d** NK cytotoxic activity (percentage of lysis of tumor cells); **e** Proliferation of lymphocytes in response to the mitogen Phytohaematogglutinin (PHA) in counts per minute (c.p.m.); **f** Concentrations (pg/mL) of IL-1β in plasma stimulate with LPS. “Placebo group” (*N* = 11) slept on normal beds; “Experimental group” (*N* = 20) used the HOGO system to avoid EMFs. a: *P < 0.05*; aa: *P < 0.01*; aaa: *P < 0.001* with respect to the values obtained at T0; b: *P < 0.05* with respect to the values obtained in "Placebo group" at T2

In order to evaluate the effects of using a bed that avoids EMFs on the immune system, the following functions of neutrophils (adherence, chemotaxis and phagocytic capacities) and lymphocytes (adherence, chemotaxis, NK activity and lymphoproliferative response to the mitogen PHA) from peripheral blood were measured. The results are shown in Fig. 2.

Neutrophil and lymphocyte chemotaxis (Fig. 2a and b) showed an increase in values (*p* < 0.01) after sleeping using the HOGO system as well as in phagocytic index (*p* < 0.05; Fig. 2c), NK cytotoxicity (*p* < 0.01; Fig. 2d) and PHA-stimulated lymphoproliferation (*p* < 0.01; Fig. 1e). IL-1β release in plasma in response to the mitogen LPS was also increased (*p* < 0.05; Fig. 1f). In the placebo group there were no statistically significant differences in any parameter before (T0) and after the 2 months (T2) of sleep on normal beds.

In addition, the results obtained for neutrophil and lymphocyte adherence, phagocytic efficacy and proliferation capacity and cytokine release of lymphocytes in basal conditions are shown in Table 2. A decreased tendency for lymphocyte adherence and for basal lymphoproliferation can be observed. However, no changes were found regarding neutrophil adherence, phagocytic efficacy and concentrations of IL-10, IL-4, IL-6 and TNF-α. In the placebo group no statistical differences were observed.

**Table 2:** Immune function parameters in peripheral blood leukocytes before (T0) and after 2 months (T2). Each value is the mean ± standard deviation of the data obtained from 31 men and women. “Placebo group” (*N* = 11) slept on normal beds; “Experimental group” (*N* = 20) used the HOGO system to avoid electromagnetic fields (EMFs). c.p.m. = counts per minute; N.S = statistically non-significant; T* = statistical tendency respect to T0

	Placebo Group			Experimental Group		
Parameters	T0	T2	*p*-value	T0	T2	*p*-value
Neutrophil Adherence (Adherence Index)	53 ± 17	51 ± 14	0.77 (N.S)	37 ± 16	45 ± 26	0.18 (N.S)
Lymphocyte Adherence (Adherence Index)	50 ± 17	48 ± 11	0.71 (N.S)	56 ± 19	35 ± 15	0.06 (*T)
Phagocytic efficacy (% neutrophil ingesting)	83 ± 16	83 ± 13	0.77 (N.S)	93 ± 7	91 ± 5	0.6 (N.S)
Basal lymphoproliferation (c.p.m.)	1115 ± 250	1281 ± 560	0.6 (N.S)	1176 ± 791	707 ± 291	0.09 (*T)
IL-10concentrations (pg/mL)	498.12 ± 229.7	111.31 ± 24.96	0.6 (N.S)	320.275 ± 90.4	190.69 ± 90.39	0.1 (N.S)
IL-4 concentrations (pg/mL)	104.22 ± 4.34	108.3 ± 5.2	0.99 (N.S)	39.67 ± 11.19	34.95 ± 19.78	0.98 (N.S)
IL-6 concentrations (pg/mL)	6574.9 ± 1301	5069.9 ± 1754.7	0.8 (N.S)	7100.2 ± 950.2	7130.1 ± 870.1	0.9 (N.S)
TNF-α concentrations (pg/mL)	1820.6 ± 596.8	2283.3 ± 1501.9	0.78 (N.S)	1069.9 ± 442.8	1088.5 ± 517.9	0.97 (N.S)

**Fig. 3:**
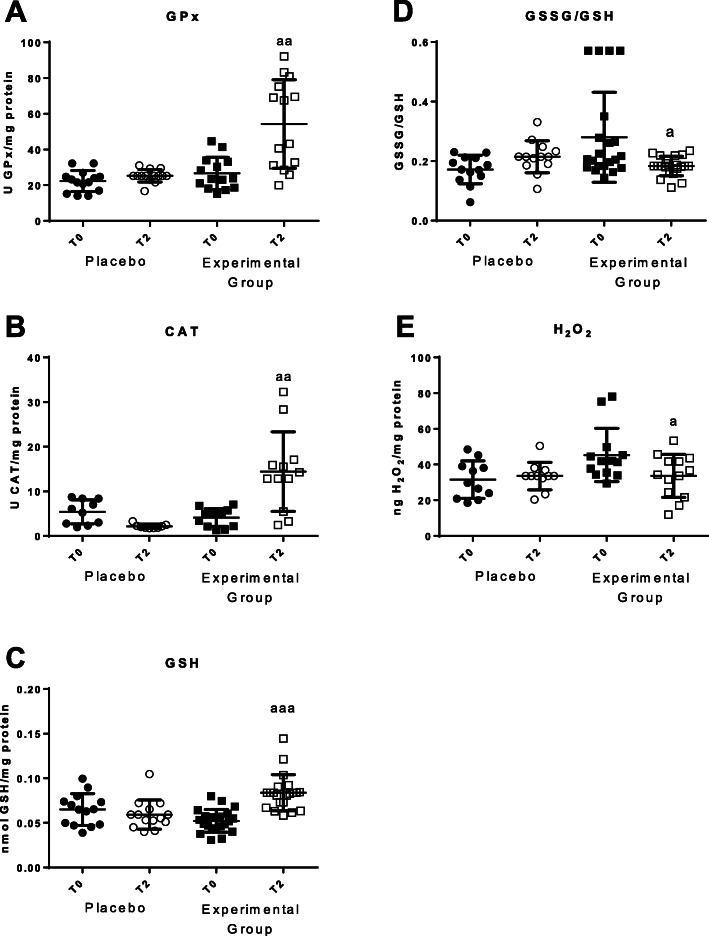
Parameters of oxidative stress in total cells blood before (T0) and after 2 months (T2). **a** Enzymatic activity of glutathione peroxidase (GPx) in U/mg protein; **b** Enzymatic activity of catalase (CAT) in U/mg protein; **c** Concentration of reduced glutathione (GSH) in nmol/mg protein; **d** GSSG/GSH ratio; **e** Concentrations of H_2_O_2_ (ng/mg protein). “Placebo group” (*N* = 11) slept on normal beds; “Experimental group” (*N* = 20) used the HOGO system to avoid EMFs; a: *P < 0.05*; aa: *P < 0.01*; aaa: *P < 0.001* with respect to the values obtained at T0

**Fig. 4:**
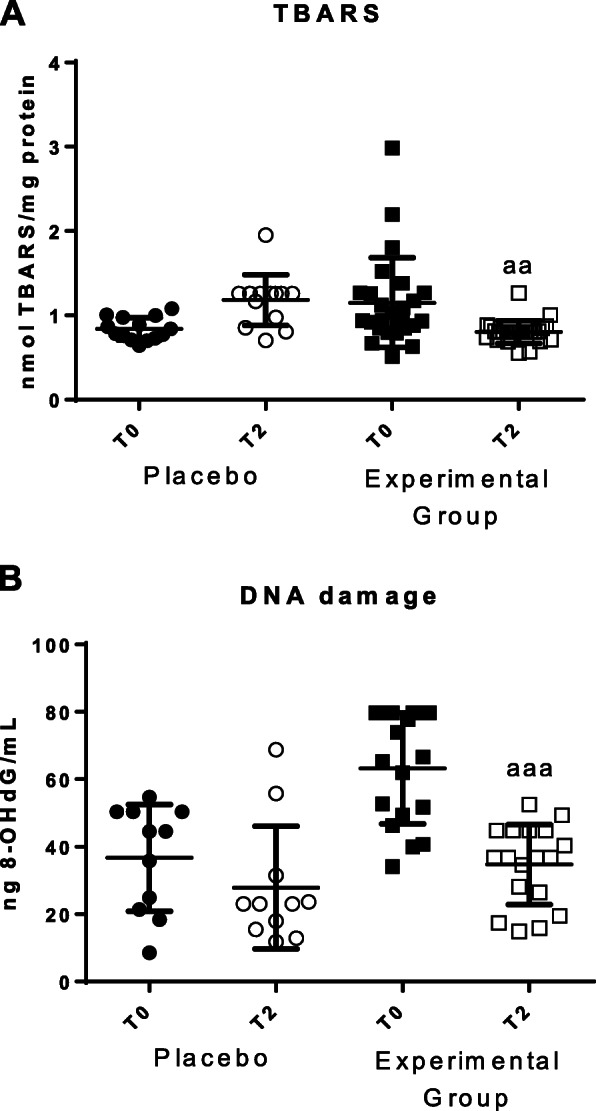
Parameters of oxidative damage in total blood cells before (T0) and after 2 months (T2). **a** Concentrations of TBARS (nmol/mg protein); **b** Concentrations of 8-oxo-2′-desoxiguanosina (8-OHdG) in ng/mL. “Placebo group (*N*=11)” slept on normal beds; “Experimental group” (*N* = 20) used the HOGO system to avoid EMFs. a: *P < 0.05*; aa: *P < 0.01*; aaa: *P < 0.001* with respect to the values obtained at T0

In total blood cells, parameters of oxidative stress such as antioxidant defenses as well as oxidants and oxidative damage to lipids and DNA were analyzed (Figs. 3 and 4). Increases in the activities of the antioxidant enzymes glutathione peroxidase (*p* < 0.01) and catalase (*p* < 0.01) (Fig. 3a and b) were observed, as well as an increase of reduced glutathione (GSH) concentration (*p* < 0.001; Fig. 3c) in T2 compared with the values at T0. Decrease concentration of the oxidant H_2_O_2_ (*p* < 0.05; Fig. 3d) was also found after the 2 months of the HOGO system usage. GSSG/GSH ratio, a marker of oxidative stress, decreased after 2 months of using the HOGO system, but only reached a statistical tendency. In the Placebo group no significant differences were observed from T0 to T2 in any of these parameters.

The values of lipid peroxidative damage (measured as amounts of TBARS) and the DNA oxidative damage (measured as 8-OH-2dG concentration), are shown in Fig. 4. A decrease of lipid peroxidation (*P* < 0.01) and of 8-OH-2dG concentrations (*p* < 0.001) after resting on the HOGO bed (Fig. 4a and b) were detected. No differences were observed in the placebo group between T0 and T2.

**Fig. 5:**
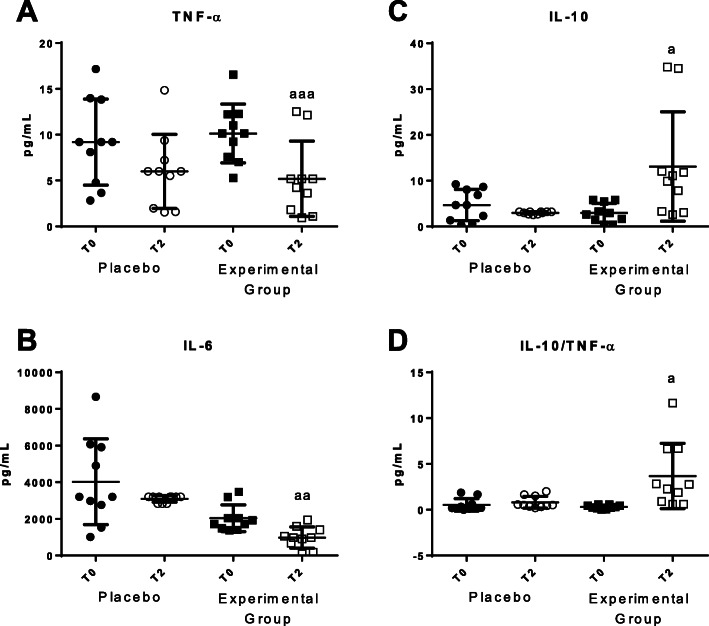
Plasmatic concentrations of cytokines released in basal conditions before (T0) and after 2 months (T2). **a** Concentrations of TNF-α (pg/mL); **b** Concentrations of IL-6 (pg/mL); **c** Concentrations of IL-10 (pg/mL) and **d** IL-10/TNF-α ratios. “Placebo group” (*N* = 11) slept on normal beds; “Experimental group” (*N* = 20) used the HOGO system to avoid EMFs. a: *P < 0.05*; aa: *P < 0.01*; aaa: *P < 0.001* with respect to the values obtained at T0

We have measured the levels of pro-inflammatory and anti-inflammatory cytokines released in basal conditions as inflammatory stress markers (Fig. 5). A statistically significant decrease of pro-inflammatory cytokines such as TNF-α and IL-6 (*p* < 0.05) were observed after using the bed protecting against EMFs (Fig. 5a and b, respectively). An increase of anti-inflammatory cytokines such as IL-10 (*P* < 0.05) was also found (Fig. 5c). The IL-10/TNF-α ratios (Fig. 5d) also increased after using the HOGO system. No statistically significant differences were detected in IL-4 and IL-1β basal values. In placebo group no differences in these parameters were observed.

**Fig. 6:**
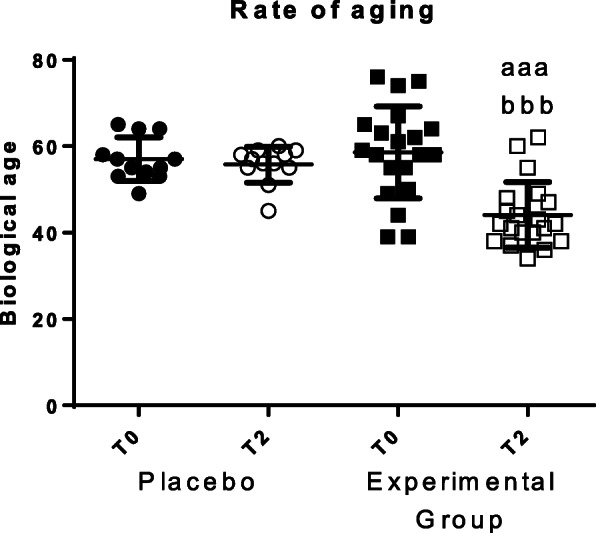
Biological age. “Placebo group” (*N* = 11) slept on normal beds; “Experimental group” (*N* = 20) used the HOGO system to avoid EMFs. a: *P < 0.05*; aa: *P < 0.01*; aaa: *P < 0.001* with respect to the values obtained at T0; bbb: *P < 0.001* with respect to the values obtained in "Placebo group" at T2

The rate of aging, or biological age, was calculated employing an equation developed using the values obtained in several immune functions [3]. The results, which are shown in Fig. 6, demonstrate that after sleeping for 2 months on the HOGO system bed, biological age decreased significantly (*p* < 0.001). The decrease of biological age was an average of 12 years. No differences were observed in the placebo group.

Finally, in order to study how sleeping on natural materials had an effect on the results obtained, immune functions were studied in a group of people who slept in beds made of non-natural materials, but with a topper that avoids the electromagnetic field. The results (Table 3) show that the participants of this group significantly improved their immune function, and their biological age with respect to their initial state.

**Table 3:** Immune functions in peripheral leukocytes before (T0) and after 2 months (T2) in “topper” group. This group of volunteers slept on beds made of non-natural materials but with a topper including the system to avoid EMFs. Each value is the mean ± standard deviation of the data obtained from 12 volunteers: 10 women and 2 men. C.I.: chemotaxis indx; c.p.m.: counts per minute; N.S: statistically non-significant; . *p < 0.05, **p < 0.01 respect to T0

Parameter	T0	T2	***p***-value
Neutrophil Chemotaxis (C.I.)	240.9 ± 137.9	415.9 ± 102.6	0.007**
Neutrophil phagocytosis (Phagocytic Index)	394.2 ± 126.2	471.6 ± 118.6	0.05*
Lymphocytes chemotaxis (C.I.)	229.2 ± 177.9	256.6 ± 138	N.S.
NK activity (% Lysis of tumoral cells)	50.36 ± 19	65 ± 18.2	0.04*
PHA-stimulated lymphoproliferation (c.p.m)	9067.7 ± 4204.9	16388.8 ± 9186.1	0.01**
Biological age	61.5 ± 8,5	49.7 ± 7.15	0.002**

## Discussion

The results of the present study suggest that the resting on a HOGO bed made using natural materials and with a system that mitigates electromagnetic fields (EMFs), was able to improve, in general, all the parameters studied both those of immune functions as well as those of oxidative-inflammatory stress, as well as to decrease the rate of aging.

Obtaining a good quality of sleep, in our current society, is becoming more and more difficult. In the framework of the bidirectional communication between the nervous and the immune systems [37], it has been observed that there is an association between sleep and the immune system [14]. Thus, an appropriate sleep has been related to an adequate immune response since sleep enhances immune defenses and sleep can also be promoted by signals produced by immune cells [14]. Moreover, in humans, sleep time becomes shorter, more fragmented, and of poorer quality with our modern life style and with advancing age [38].

Currently the use of objects which increase our exposure to EMFs has increased exponentially. Although there are contradictory results, EMFs largely seem to induce damage in humans [20, 21, 25]. The majority of studies show the relation between EMFs and the risk of many pathologies, especially cancer and neurological diseases, amongst others [39]. Moreover, exposure to EMFs seems to produce impairment in sleep as well as to generate fatigue and general discomfort [40]. In fact, since the immune system is a good marker of health [1, 2], and the EMFs can produce alterations in this system [41], our exposure to these could result in an impairment of health. A possible pathway could be the increase in oxidative stress and inflammation that the EMFs produce in our cells [42]. Thus, following the idea that oxidative-inflammatory stress is the base of aging [2], the daily exposure to EMFs could promote an acceleration of the aging process. In spite of this the effects of mitigating EMFs on the immune system, redox and inflammatory states, during the nocturnal sleep period, have not yet been studied.

In the present work, several functions have been studied in isolated leukocytes as well as parameters of oxidative stress in total blood cells and inflammatory markers in plasma, before and after 2 months of sleep on EMF mitigating HOGO beds. The results of the present study found that sleep using this kind of bed, was able to improve all the immune cell function parameters studied. Thus, innate immunity functions such as chemotaxis, phagocytic capacity and Natural Killer activity as well as the lymphoproliferation in response to mitogens, an adaptive immunity activity, were increased. These functions decrease, in both humans and mice, with chronological aging [3], but also in states of premature and accelerated aging [42]. In the case of mice, in which the values of these functions can be associated with individual life span, it has been observed that they are heavy determinants of the longevity of each subject [43]. Several lifestyle strategies aim to improve these functions in humans and mice [7]. In the case of mice, in which it is possible to record the longevity, this improvement is followed by an increase in life span [7]. We have a mathematic model, using these functions, to determine the biological age of a person [3]. As previously mentioned EMFs exposure is linked to rapid aging, reducing longevity from 80 years to 65–70 years [20]. The application of this model in the volunteers of the present study showed that after sleeping on the HOGO system, mitigating EMFs, there was a rejuvenation of the biological age. This slowdown of the rate of aging was appreciated in all the participants with an average of 12 years.

With respect to the release of cytokines in the presence of a mitogen, a function that decreases, in general, with aging [44], the results showed an increase in IL-1β after using this system. It is known that in response to a pathogen, immune cells have to produce and release higher amounts of pro-inflammatory cytokines to allow an adequate defense [2, 45]. Our study demonstrated improvements in these functions for human participants after employing the HOGO system.

The oxidative stress (higher production of oxidants in relation to the antioxidant defenses) is the base of immunosenescence and aging [2]. Thus, with the advance of age there is an increase of oxidants and a decrease of antioxidants [46]. This age-related oxidative stress leads to oxidative damage of biomolecules such as lipids and DNA [40]. In the case of DNA, when this is modified by oxidative damage, it produces 8-OH-dG [47].

Longevity is related to an individual’s oxidative stress rate [36]. Several life style strategies have been shown to decrease this oxidative stress and consequently increase the health and longevity [7]. EMFs seem to result in oxidative stress [48, 49] and oxidative damage to lipids [50]. In fact, some studies have observed that GPx [51] and CAT [52] activities were decreased in the long-term EMF exposure group. Our results show an increase of GPx and CAT activities after sleeping on an EMF insulating system. Moreover, we also observed a significant decrease in oxidative compounds such as H_2_O_2_ and GSSG. Consequently, the oxidative damage, both in lipids (MDA) and DNA (8-OH-dG) were also decreased.

In addition, it is known that sleep quality may buffer age-related increases in state of inflammation [16]. EMFs can promote inflammatory state, increasing pro-inflammatory cytokines such as IL-6 and TNF-α [25, 53, 54]. Moreover, EMF exposure 2 h/day for 1 month produces a decrease of IL-4 production in rat serum [55]. Thus, sleeping on an EMF avoiding system decreases the levels of these pro-inflammatory cytokines and increases the anti-inflammatory cytokine IL-10, released in basal conditions.

Since in humans, in the framework of neuroimmune communication, the placebo effect of a strategy is possible [56], in the present study we analyze this possibility. Interestingly, volunteers that slept in a bed similar to the experimental system, but without any of its characteristics, did not show any change in the parameters measured.

All these results support the ability of the HOGO system to decrease the rate of aging, i.e., the biological age of the volunteers investigated in the present study. We do not know the direct causes of this effect, and further studies are needed to clarify this subject. Nevertheless, we think that although the use of natural materials in these beds can help, the positive effects observed are due to the avoidance of EFMs. This idea can be deduced from the positive results obtained in the “topper” group, which used the avoiding EMFs system without the rest of the natural components of the complete bed.

Nevertheless, this study presents some limitations. Besides the above commented, one of the limitations is the sample size used in this work. Although to increase the number of participants is very expensive due to the cost of beds, with more volunteers it would be possible to divide volunteers into different groups and to establish correlations with characteristics of profession, social class, as well as to take into account the effect of different environmental factors (such as room light, noise...). Another limitation is that the long-term effect of the strategy used in the present study has not been investigated and could be interesting to check whether after a year, for example, the maintenance of a younger biological age occurs.

With these limitations in mind, the present study provides evidence for an effective and non-intrusive EMF mitigating strategy, which positively affects several immune functions and oxidative-inflammation markers and opens new avenues for promoting a healthier longevity.

## Conclusions

Sleeping on beds with an EMFs-mitigating system improves the functionality of the immune system as well as reduces the oxidative and inflammatory stress. Finally, sleeping on beds with an EMFs-mitigating system slows down the rate of aging.

## Data Availability

All data generated or analyzed during this study are included in this published article.

## Abbreviations

EMF: Electromagnetic field
DNA: Deoxyribonucleic acid
NK cells: Natural Killer cells
ROS: Reactive oxygen species
Hz: Hertz
KHz: Kilohertz
MHz: Megahertz
GHz: Gigahertz
nT: Nanotestla
mV: Milivolt
v/m: Volt/metre
μW/m^2^: Microwatt/square metre
LDH: Lactate deshydrogenase
PHA: Phytohaemagglutinin
c.p.m.: Counts per minute
RPMI medium: Medio Roswell Park Memorial Institute medium
CAT: Catalase
U: Units
mL: Millilitre
mg: Milligram
pg: Pictogram
ng: Nanogram
GPx: Glutathione peroxidase
NADPH: Nicotinamide adenine dinucleotide phosphate
EDTA: Ethylenediaminetetraacetic acid
GSH: Reduced glutathione
GSSG: Oxidized glutathione
OPT: O-phthalaldehyde
H_2_O_2_: Hydrogen peroxide
MDA: Malondialdehyde
TBA: Thiobarbituric acid
TBARS: Thiobarbituric Acid Reactive Substances
ELISA: *Enzyme-Linked ImmunoSorbent Assay*
8-OHdG: 8-hydroxy-2′-deoxyguanosine
LPS: Lipopolysaccharide
IL: Interleukin
TNF: Tumor necrosis factor
T0: Initial time
T2: After sleeping on normal beds or on HOGO beds
CI: Chemotaxis index
PI: Phagocytic index
